# Eco-friendly sonochemical synthesis of BaTiO_3_/Ag nanocomposite particles: Investigation of Ag-nanoparticle formation and growth behavior under ultrasound irradiation

**DOI:** 10.1016/j.ultsonch.2025.107539

**Published:** 2025-08-28

**Authors:** Tatsuya Shishido, Yamato Hayashi, Hirotsugu Takizawa, Minoru Ueshima

**Affiliations:** a Graduate School of Engineering, Department of Applied Chemistry, Tohoku University, 6-6-07 Aoba, Aramaki, Aoba-ku, Sendai 980-8579, Japan; b Daicel Corporation, Grand Front Osaka Tower-B, 3-1, Kita-ku, Osaka 530-0011, Japan

**Keywords:** BaTiO_3_/Ag nanocomposite particle, Eco-friendly synthesis, Ultrasound, Silver oxide, Methanol

## Abstract

This study synthesizes BaTiO_3_/Ag nanocomposite particles using an eco-friendly sonochemical method and investigates the mechanism of Ag-nanoparticle deposition. BaTiO_3_ and Ag_2_O were ultrasonically irradiated in methanol at 20 ˚C, and samples and solvents were characterized using X-ray diffraction, scanning electron microscopy, scanning transmission electron microscopy with energy-dispersive X-ray spectroscopy, ultraviolet–visible spectroscopy, Brunauer-Emmett-Teller method, X-ray photoelectron spectroscopy and gas chromatography-mass spectrometry. Nanocomposite particles uniformly decorated with fine Ag nanoparticles were obtained via ultrasonication (>45 kHz) for 3 h. The average size of the Ag nanoparticles increased with increasing irradiation time, which was presumed to be due to the thermal and physical effects of the ultrasound. Even at 10 vol% Ag loading, nanocomposites exhibited uniform particle distribution. Ultrasound irradiation produced nanocomposite particles with finer Ag nanoparticles and accelerated the reduction of Ag_2_O compared with mechanical stirring. In this deposition reaction, Ag_2_O was presumed to be reduced by methanol and its radical derivatives, and BaTiO_3_ exhibited catalytic activity for Ag_2_O reduction. This study not only contributes to the understanding of the mechanism of Ag-nanoparticle deposition in alcohol-Ag_2_O systems but also offers a promising room-temperature and waste-reducing synthesis route for nanocomposite particles toward a sustainable society.

## Graphical abstract

**Figure:**
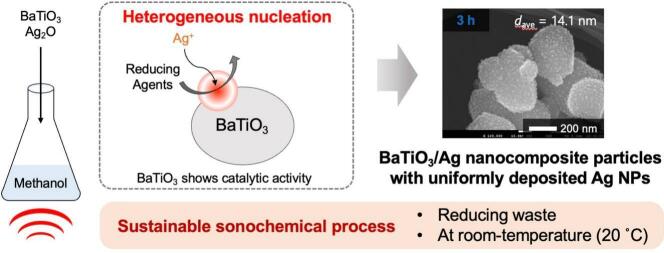

## 1 Introduction

Barium titanate/silver (BaTiO_3_/Ag) nanocomposites have attracted considerable attention due to their unique combination of properties derived from both components: the photocatalytic and dielectric characteristics of barium titanate, and the excellent thermal and electrical conductivities of silver. Silver nanoparticles show surface plasmon resonance (SPR) in addition to these properties. Owing to these synergistic features, BaTiO_3_/Ag nanocomposites have been applied in various forms such as powders and bulk and used for photocatalysts, capacitors, thermal management materials, sensors and energy device [[1], [2], [3], [4], [5], [6], [7]].

To improve the properties of these materials, the particle size and loading amount of the Ag NPs must be controlled. Liquid-phase processes such as the polyol method, reduction, and chemical-precipitation are mainly used because of their ease of control. The conventional processes and their characteristics are summarized in Table S1. In these processes, silver nitrate (AgNO_3_) is used as the precursor for the Ag NPs. In the polyol and chemical-precipitation methods, dispersants (polyvinylpyrrolidone) and pH adjusters (sodium hydroxide) are used. Because of the use of these raw materials, ion species (Na^+^, NO_3_^–^, etc.) and dispersants remain after synthesis. Their removal requires a washing process, which increases solvent consumption and consequently generates more waste. Additionally, the polyol method requires a high-temperature reaction above 100 ˚C. Thus, conventional processes are limited, and a new, low-environmental-impact process is required.

To address these environmental issues, we explored a sonochemical process with Ag_2_O as an eco-friendly raw material. Unlike AgNO_3_, Ag_2_O does not leave a residual counter anion after the reaction and has been reported to be reduced to Ag under ultrasound irradiation in alcohol, which is relatively safe and inexpensive [8]. The sonochemical process utilizes the cavitation generated by ultrasound irradiation in the liquid phase. The collapse of cavitation generates a high-temperature and high-pressure hotspot for a very short duration at the microsecond scale. These hotspots promote chemical reactions and nucleation [9,10]. Therefore, the combination of Ag_2_O with the effects of the sonochemical process enables the waste-reducing synthesis of nanocomposite particles at room temperature. We successfully synthesized noble metal NPs, alloy, various nanocomposite particles, and Ag NP-supported films, such as Ag NPs, Au-Ag alloy, BaTiO_3_/Ag, multi-walled carbon nanotube/Ag, graphene/Ag, glass/Ag, and Cu/Ag, by applying ultrasound irradiation to Ag_2_O in ethanol [[11], [12], [13], [14], [15], [16]]. This process is highly generalizable and applicable to the synthesis of a wide range of materials with an almost quantitative yield. Moreover, the loading amount can be easily controlled by simply adjusting the amount of Ag_2_O added, and fine Ag nanoparticles can be supported even at high loadings. Therefore, this process holds potential for application as a precursor for capacitors, conductive films and energy device, where fine microstructures are required to enhance material properties [5,11,12]. In addition to the advantages mentioned above, this process, unlike previous ones, does not require complex operations and is therefore expected to be easier to scale up [17]. However, silver acetate was generated as a byproduct of the use of ethanol. In most previous studies, only a single frequency (45 kHz) was used; therefore, investigations of the effects of ultrasound are insufficient.

In this study, BaTiO_3_/Ag nanocomposite particles are synthesized using methanol as the solvent at room temperature. Nanocomposite particles with different Ag volume fractions are synthesized for various applications. The continued ultrasound irradiation led to the formation of nanocomposite particles with different Ag NP size. In addition, we discuss the effects of ultrasound frequencies and mechanism of Ag-NP deposition based on the ultrasound frequency and mechanical stirring.

## 2 Experimental

### 2.1 Chemicals & equipment

Ag_2_O (99.0 %, Fujifilm Wako Pure Chemical Corporation, Japan) and BaTiO_3_ (BT-03, Sakai Chemical Industry Co., Ltd., Japan) were used as starting materials. Methanol (99.5 %, Fujifilm Wako Pure Chemical Corporation, Japan) and ethanol (99.0 %, Japan Alcohol Trading Co., Ltd., Japan) were used as the solvent. SiO_2_ (Sciqas, 0.4 µm, Sakai Chemical Industry Co., Ltd., Japan) was used for control experiment. All chemicals were used as received without further purification.

Ultrasound treatment was performed using a sonoreactor system (Honda Electronics Co., Ltd., Japan). A schematic of the operating system is shown in Fig. S1. The irradiation device was a transducer that applied ultrasound indirectly to an Erlenmeyer flask placed in a water bath. Thus, some of the irradiated energy is absorbed by the water bath and the flask container. The effective absorbed acoustic power *P*_ac_ [W] was determined by calorimetry [18]. Cavitation intensity was measured using a sonic monitor (HUS-5, Honda Electronics Co., Ltd., Japan). This device uses a quartz-glass probe to evaluate cavitation intensity as a relative current value. The input power, cavitation intensity and energy efficiency at each frequency are summarized in Table S2. The temperature of the water bath was controlled using a circulating cooling device (CTP-3000; Tokyo Rikakikai Co., Ltd., Japan).

A laboratory stirrer (LM200, Yamato Scientific Co., Ltd., Japan) was used for the mechanical-stirring experiments.

### 2.2 Preparation of BaTiO_3_/Ag nanocomposite particles

A 300 mL Erlenmeyer flask was filled with 100 mL methanol and degassed by ultrasound irradiation (45 kHz, *P*_ac_ = 11 W) for 30 min. Subsequently, BaTiO_3_ and Ag_2_O were added, and the flask was irradiated in a water bath at 20 ˚C for 1–24 h. The effects of frequency were investigated at 24, 45, and 100 kHz while maintaining an effective power of 11 W for all experiments. The Ag volume fractions (Ag/(BaTiO_3_ + Ag) = 1, 3, 5, and 10 vol%) were examined at 45 kHz. Table 1 lists the experimental conditions used in the study.

**Table 1:** Experimental conditions for the synthesis of BaTiO_3_/Ag nanocomposite particles.

No.	BaTiO_3_ (g)	Ag_2_O (g)	Ag (vol%)	Frequency (kHz)
1	1.00	0.0976	5	24
2	1.00	0.0976	5	45
3	1.00	0.0976	5	100
4	1.00	0.0187	1	45
5	1.00	0.0573	3	45
6	1.00	0.2059	10	45

The procedure for the mechanical-stirring experiment was as follows. Methanol (100 mL) was added to a 300 mL Erlenmeyer flask and degassed. Subsequently, BaTiO_3_ and Ag_2_O (Ag 5 vol%) were added, followed by mechanical stirring at 200 rpm in a 20 °C water bath for 1–24 h. Experiments with only Ag_2_O (0.0976 g) under mechanical stirring were also conducted.

After ultrasound irradiation or mechanical stirring, BaTiO_3_/Ag nanocomposite particles were obtained by vacuum-drying the dispersion overnight.

### 2.3 Characterization

The crystalline phases of the samples were determined by X-ray diffraction (XRD, D2 Phaser, Bruker AXS, Germany). Measurements were performed using Cu Kα radiation (λ = 0.154184 nm) over a 5°–80° range. The morphologies of the samples were characterized using field-emission scanning electron microscopy (FE-SEM; JSM-7610F, JEOL Ltd., Japan). The particle-size distributions of the Ag NPs were obtained by analyzing the SEM images using ImageJ, a free image-analysis software. Elemental mapping was performed using scanning transmission electron microscopy with energy-dispersive X-ray spectroscopy (STEM-EDX, HD-2700, Hitachi High-Tech Corp., Japan). The diffuse reflectance spectra of the samples were measured in the range of 200–800 nm using ultraviolet–visible (UV–vis) spectroscopy (V-750, JASCO Corp., Japan). Diffuse reflectance spectra were converted to absorption spectra using the Kubelka–Munk function. The absorption spectra of the solvents after ultrasound irradiation were also measured. The specific surface areas (*S*_BET_) of BaTiO_3_ and BaTiO_3_/Ag nanocomposite particles were measured using a catalyst characterization analyzer (BELCAT II, MicrotracBEL Corp., Japan). The solvent was analyzed using gas chromatography–mass spectrometry (GC–MS; Agilent 8860, 5977B, Agilent Technologies Inc., USA). The surface state of BaTiO_3_ was analyzed by X-ray photoelectron spectroscopy (XPS, Theta Probe, ThermoFisher Scientific, America). The reaction ratio of Ag_2_O measured by thermogravimetry–differential thermal analysis (TG-DTA; STA-2500, NETZSCH, Germany). Under an N_2_ flow, the sample was heated to 500 °C at a heating rate of 20 °C min^−1^, and the weight loss due to the thermal decomposition of silver oxide was used for the calculation.

## 3 Results & discussion

### 3.1 Effects of irradiation time

**Fig. 1:**
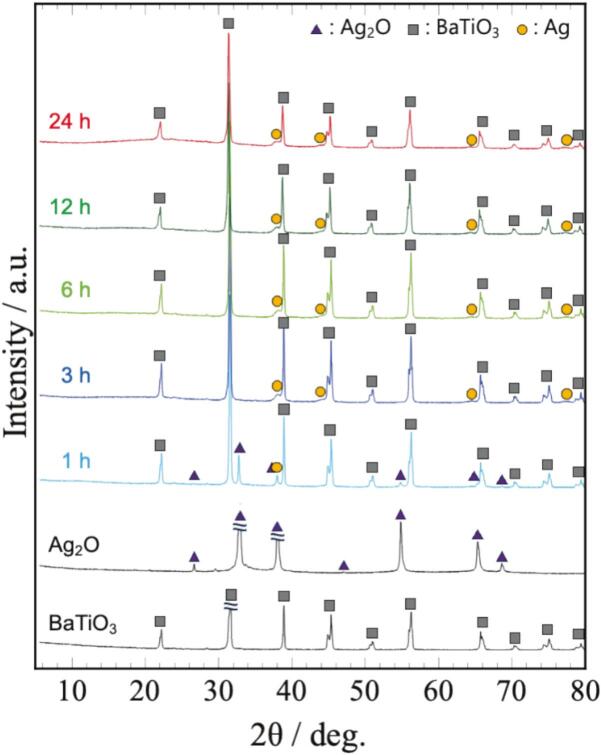
XRD patterns of starting materials and samples prepared by 45 kHz irradiation.

Fig. 1 shows the XRD patterns of the starting materials and samples prepared by 45 kHz irradiation for 1–24 h (Sample No.2). The samples exhibited peaks corresponding to Ag, indicating that Ag_2_O was reduced to Ag by ultrasound irradiation in methanol. The Ag_2_O peaks disappeared after 3 h of irradiation. This result indicates that the synthesis of the nanocomposite particles was completed within 3 h.

**Fig. 2:**
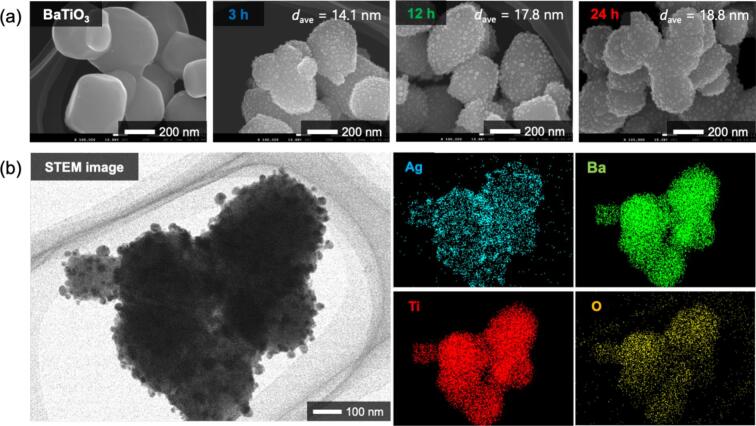
(a) SEM images and (b) element mapping of samples synthesized by 45 kHz irradiation.

The SEM images of the BaTiO_3_ particles and synthesized samples are shown in Fig. 2 (a). BaTiO_3_ particles were about 300 nm and had smooth surface. After ultrasound irradiation, Ag NPs were deposited on the BaTiO_3_ surface. The particle-size distribution of the Ag NPs (Fig. S2 and Table S3) showed that the average particle size of the Ag NPs was 14.4 nm after 3 h of irradiation and increased to 18.8 nm after 24 h. Based on the XRD pattern shown in Fig. 1, the reduction was complete after 3 h of irradiation, suggesting that the Ag NPs grew without the Ag source (Ag_2_O). As shown in the elemental-mapping images in Fig. 2 (b), the Ag mapping remained highly uniform after 24 h. This result revealed that nanocomposite particles with uniformly dispersed Ag NPs were obtained. In our previous study, nanocomposite particles were synthesized in ethanol at 40 ˚C, and silver acetate was also obtained as a byproduct [14]. However, in this study, we successfully synthesized BaTiO_3_/Ag nanocomposite particles at a lower temperature (20 ˚C) without waste or byproducts. The *S*_BET_ of BaTiO_3_ and BaTiO_3_/Ag nanocomposite particles (3 h) were 2.9 and 3.8 m^2^/g, respectively. These values are comparable to those of conventional photocatalysts [19], indicating that the synthesized BaTiO_3_/Ag nanocomposite particles may have potential for photocatalytic applications.

We also observed the morphology of Ag_2_O. Ag_2_O had agglomerated particles of approximately 3–5 µm, and the surface of the residual Ag_2_O was eroded after 1 h of irradiation (Fig. S3).

### 3.2 Effects of ultrasound frequency

**Fig. 3:**
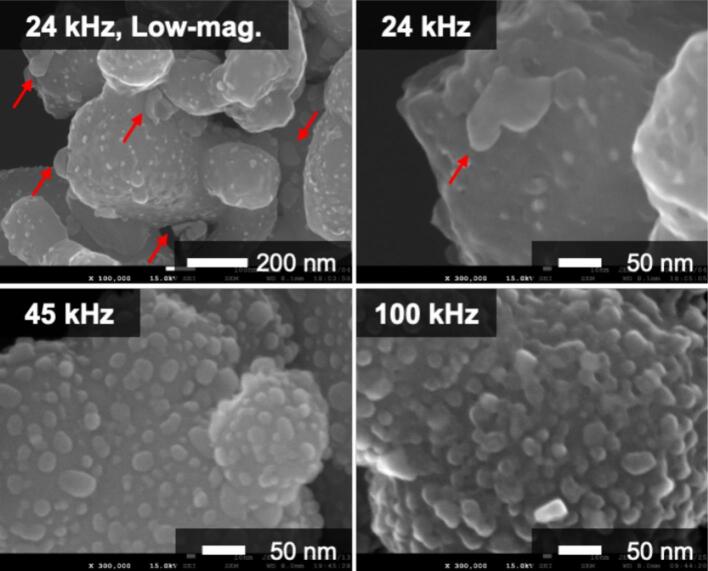
SEM images of 3 h samples prepared using 24, 45, and 100 kHz irradiation. The image of the 45 kHz irradiation is a high-magnification image of (a).

To investigate the effect of frequency on the morphology of the nanocomposite particles, they were synthesized at three different frequencies: 24, 45, and 100 kHz (Sample No.1–3). Based on the XRD patterns (Fig. 1 and Fig. S4), Ag_2_O was reduced to Ag by ultrasound irradiation in methanol. The reduction of Ag_2_O was completed after 3 h of irradiation, indicating that the ultrasound frequency did not significantly influence the reduction rate. Fig. 3 shows the SEM images of the samples prepared at different ultrasound frequencies. In the sample prepared by 24 kHz irradiation, Ag NPs were not uniformly deposited on the BaTiO_3_ particles, and agglomerated Ag NPs (∼100 nm) were observed (highlighted by the arrow in the SEM image). In contrast, the agglomeration of Ag NPs was not observed, and nanocomposite particles with uniformly deposited Ag NPs were synthesized at frequencies greater than 45 kHz. In particular, Ag NPs were tightly supported and necked at 100 kHz for 3 h. Similar to the case with 45 kHz, long-duration ultrasound irradiation resulted in the growth of Ag NPs, and after 24 h, the necking disappeared, leading to the formation of spherical Ag NPs (Fig. S5). The above results show that ultrasound irradiation at 45 and 100 kHz was suitable for the fine and uniform deposition of Ag NPs. Moreover, the morphology of the Ag NPs changed owing to the ultrasound irradiation.

### 3.3 Investigation of Ag volume fraction

**Fig. 4:**
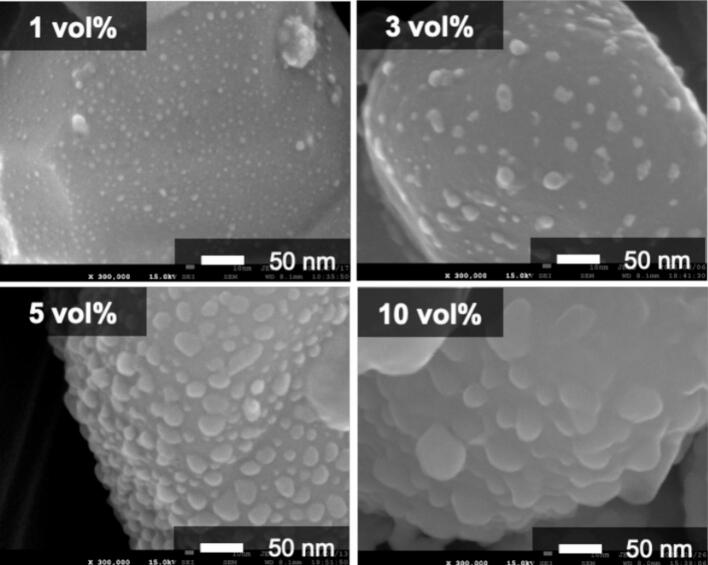
SEM images of samples with different Ag volume fractions (3 h sonication).

We synthesized various BaTiO_3_/Ag nanocomposite particles with different Ag volume fractions (1–10 vol%) considering their potential for various applications (Sample No.2, 4–6). At 1 vol%, the amount of added Ag_2_O was extremely low; therefore, no peaks corresponding to Ag_2_O or Ag were observed in the XRD pattern for up to 12 h of irradiation (Fig. S6 (a)). After 24 h of irradiation, only the strongest peak corresponding to metallic Ag was observed. In contrast, for the samples with Ag volume fractions of 3, 5, and 10 vol%, the Ag_2_O peaks disappeared after 3 h of irradiation, indicating the completion of Ag NP deposition (Fig. 1, Fig. S6 (b, c)). The SEM images shown in Fig. 4 indicate that Ag NPs were deposited at all Ag volume fractions. At 1 vol%, extremely fine Ag NPs with single-nanometer sizes were deposited. As the Ag volume fraction increased, the size of the Ag NPs increased. Even with 10 vol% loading, uniform BaTiO_3_/Ag nanocomposites with 30–50 nm Ag NPs were synthesized.

**Fig. 5:**
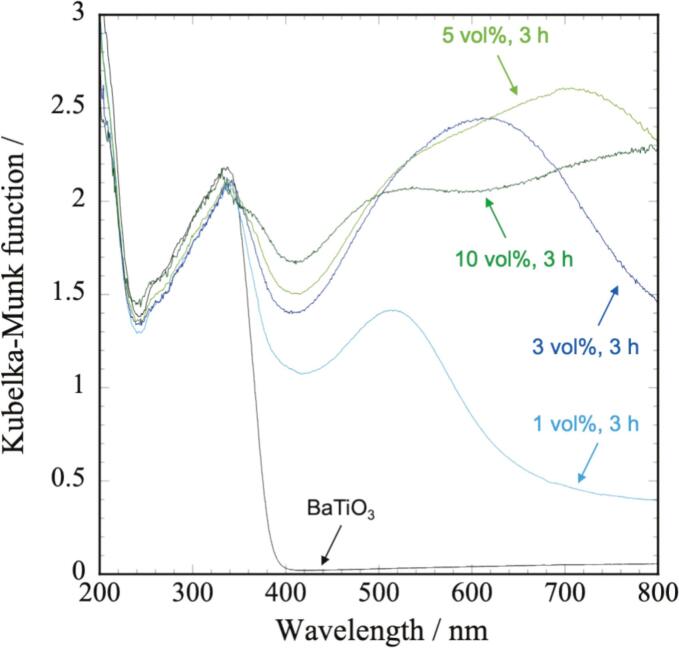
UV–vis spectra of BaTiO_3_/Ag nanocomposite particles.

Photocatalysis is a potential application of BaTiO_3_/Ag nanocomposites, in which UV–vis absorption is a key characteristic. Fig. 5 shows the UV–vis spectra of the nanocomposites prepared with different Ag volume ratios. While pure BaTiO_3_ did not exhibit absorption in the visible-light region, the BaTiO_3_/Ag nanocomposite particles exhibited absorption above 400 nm. This absorption was considered to originate from the SPR of the Ag NPs. The absorption wavelengths were 513, 610, and 706 nm for 1, 3, and 5 vol%, respectively, which are significantly different from the typical SPR of Ag NPs (approximately 400–420 nm) [[20], [21], [22], [23]]. In addition, the absorption peaks broadened as the Ag volume fraction increased, and no distinct absorption peak was observed at 10 vol%, unlike in the 1–5 vol% range. The reason for this large red-shift is not well understood, but the particle size and loading density of Ag NPs, along with the influence of BaTiO_3_, are presumed to be the contributing factors. It is well known that the SPR peak of Ag NPs exhibits red-shift as the particle size increases [22]. According to the plasmonic coupling theory, decreasing the interparticle distance between Ag NPs leads to a red shift in their SPR peak [24]. As the Ag loading increases, the particle size of Ag NPs becomes larger and their loading density increases, resulting in a shorter interparticle distance. Consequently, it is considered that the nanocomposite particles with higher Ag loading showed the larger red-shift. Furthermore, the SPR peak position of Ag NPs is influenced by the refractive index of the surrounding medium; a higher refractive index results in a larger red-shift [21]. Since the nanocomposite particles were synthesized without a dispersant, strong interactions between the Ag NPs and BaTiO_3_ are expected. Given that BaTiO_3_ has a high refractive index (*n* = 2.4), the SPR peak is presumed to have red-shifted accordingly.

### 3.4 Discussion of mechanism of Ag NP deposition on BaTiO_3_ particles

**Fig. 6:**
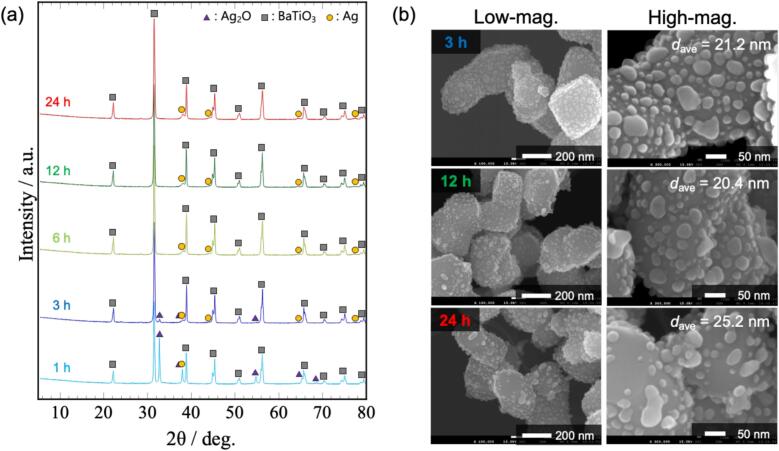
(a) XRD patterns and (b) SEM images of samples prepared by mechanical stirring.

To investigate the effects of ultrasound, the nanocomposite particles were synthesized by mechanical stirring. Fig. 6 (a) shows the XRD patterns of the samples prepared by mechanical stirring. Ag_2_O was reduced to metallic Ag when Ag_2_O and BaTiO_3_ were stirred in methanol. After 6 h of irradiation, the Ag_2_O peak was no longer observed. Therefore, ultrasonic irradiation led to a faster reduction of Ag_2_O. The SEM images of the samples are shown in Fig. 6 (b). Ag NPs were deposited on the surface of BaTiO_3_; however, the average size of the Ag NPs was larger (>20 nm) than in the case of ultrasound irradiation (18.4 nm after 24 h of irradiation). Aggregated Ag NPs with nonspherical shapes were also observed. Thus, mechanical stirring resulted in the synthesis of nanocomposite particles with nonuniformly deposited Ag NPs.

A more detailed comparison between ultrasound irradiation and mechanical stirring was conducted. The reaction rate of Ag_2_O was evaluated using TG-DTA (Fig. S8(a)). Assuming a first-order reaction, linear fitting yielded good agreement, and the reaction rate constants for ultrasound irradiation and mechanical stirring were determined to be 0.0209 and 0.0073 min^−1^, respectively (Fig. S8(b)). Therefore, it was revealed that synthesis under ultrasound irradiation exhibited a reaction rate approximately 2.9 times higher than that under mechanical stirring.

The above results indicate that ultrasound irradiation not only accelerates Ag_2_O reduction, but also contributes to the size reduction and uniform deposition of Ag NPs. However, the results of the mechanical-stirring experiments showed that the reduction of Ag_2_O occurred in the absence of ultrasound irradiation. To further verify this result, mechanical stirring of Ag_2_O was conducted and the reduction of Ag_2_O did not progress significantly after 24 h of stirring (Fig. S7). Therefore, the reduction of Ag_2_O was promoted in the presence of BaTiO_3_.

**Fig. 7:**
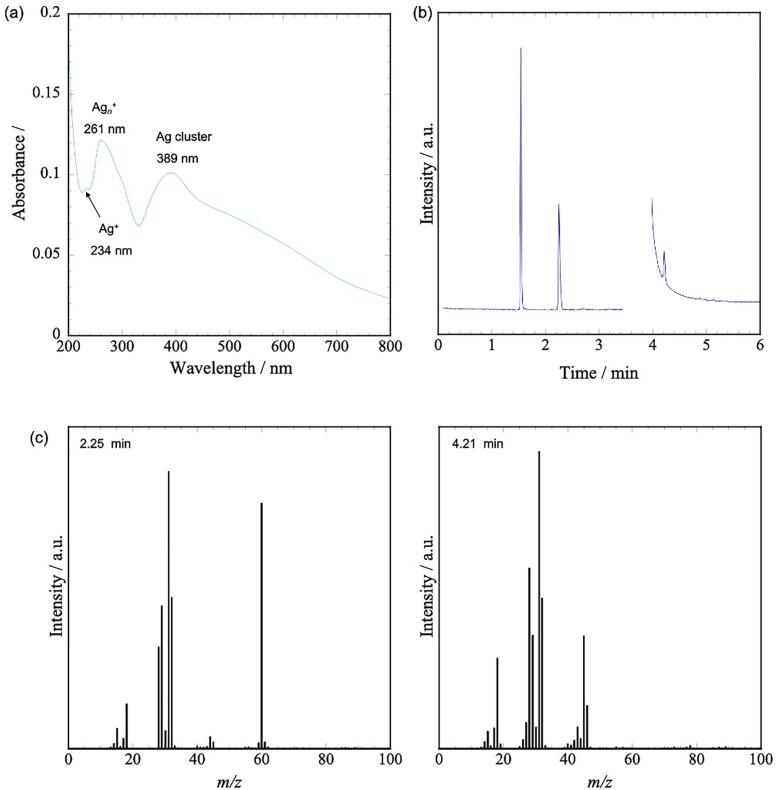
(a) UV–vis spectra of solvent after 1 h irradiation. (b) GC of solvent after 3 h irradiation (45 kHz, 5 vol%). (c) Mass spectra at 2.25 min and 4.21 min.

After the synthesis, the solvents were analyzed to further understand the mechanism of Ag-NP deposition on BaTiO_3_. Fig. 7 (a) shows the UV–vis spectrum of the solution after 1 h of irradiation. Absorption peaks were observed at 234, 261, and389 nm, which correspond to Ag^+^, Ag*_n_*^+^ (Ag ion cluster), and Ag*_n_*^0^ (Ag clusters), respectively [[25], [26], [27], [28]]. This result indicates that Ag^+^ and Ag*_n_*^+^ dissolved in methanol from Ag_2_O. Ag clusters were presumed to be formed by the reduction of Ag^+^ and Ag*_n_*^+^ in the solution (homogeneous nucleation) and by the detachment of metallic Ag due to the physical effects of ultrasound from the surface of BaTiO_3_ or Ag_2_O. Fig. 7 (b) and (c) show the GC–MS results of the solvents after 3 h of irradiation. In the chromatogram shown in Fig. 7(b), peaks are observed at 1.54, 2.25, and 4.21 min, which were attributed to air, methyl formate, and ethanol, respectively, based on the MS spectrum in Fig. 7(c). The methanol peak appearing between 3.46 and 3.95 min was not recorded to protect the GC–MS detector.

**Fig. 8:**
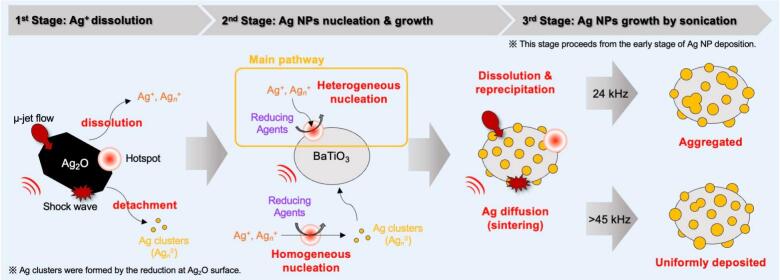
Schematic of mechanism of Ag-NP deposition on BaTiO_3_ particles.

Based on previous results, the mechanism of Ag-NP deposition on the BaTiO_3_ surface is discussed. A schematic of this process is shown in Fig. 8.

First, the Ag precursors were released from the Ag_2_O microparticles (1st Stage). Ag^+^ and Ag*_n_*^+^ dissolved in the solution from the surface of Ag_2_O (Fig. 7 (a)). In the first stage, ultrasound irradiation activated the surface of Ag_2_O [[29], [30], [31]] and enhanced the dissolution of Ag^+^ and Ag*_n_*^+^ [[32], [33], [34]]. Furthermore, the uniform nucleation of the Ag precursor at hotspots resulted in the formation of Ag clusters. In addition, metallic Ag was formed by reduction at the surface of Ag_2_O. Ag clusters were also formed by detachment of this metallic Ag from the surface due to the physical effects of ultrasound.

In the second stage, the heterogeneous nucleation and growth of Ag NPs on the BaTiO_3_ surface occurred via the reduction of the Ag precursor and deposition of Ag clusters. Ag^+^ and Ag*_n_*^+^ were adsorbed onto the BaTiO_3_ surface and reduced using reducing agents, leading to the deposition of Ag NPs. Generally, Ag clusters are synthesized and stabilized in the presence of dispersants [[35], [36], [37], [38]]. Therefore, the Ag clusters presented in this study are expected to be unstable in the absence of such dispersants. Thus, Ag clusters were supported on the BaTiO_3_ surface. As these phenomena were repeated, the nucleation and growth of Ag NPs proceeded. Compared with the mechanical-stirring experiment (Fig. 6), ultrasound irradiation (>45 kHz) accelerated the reduction of Ag_2_O and led to the uniform and fine deposition of Ag NPs. This result suggests that the high-temperature field generated by cavitation promotes both reduction and nucleation [[39], [40], [41]].

Based on the conditions of the first and second stages, two possible routes for the loading of Ag NPs are proposed: one through reduction on the BaTiO_3_ surface and the other through the deposition of Ag clusters generated in the solution or by detachment from Ag_2_O. During the mechanical-stirring experiments (Fig. S7), the reduction of Ag_2_O barely occurred. This result indicates that the reduction of Ag_2_O was promoted by the addition of BaTiO_3_. In general, the Gibbs free energy of heterogeneous nucleation is lower than that of homogeneous nucleation, making heterogeneous nucleation more likely [42]. To investigate the effect of an increased number of nucleation sites, we examined the loading of Ag NPs onto amorphous SiO_2_ particles. When SiO_2_ particles were added in an amount corresponding to the surface area of BaTiO_3_, the reduction time of Ag_2_O was prolonged to 12 h (Fig. S9). This result indicates that factors other than simply increasing the number of nucleation sites contributed to the acceleration of Ag_2_O reduction. Previous reports have shown that BaTiO_3_ exhibits catalytic activity in the reduction of Ag^+^ [43]. When BaTiO_3_ was added to an ethylene glycol solution containing dissolved AgNO_3_, Ag^+^ ions were reduced at room temperature. This reaction likely progresses because of the catalytic role of the oxygen vacancies in BaTiO_3_. XPS measurements were also conducted for BaTiO_3_ used in this study (Fig. S10). From the Ti 2p spectrum, two chemical species, Ti^4+^ (458.8 eV) and Ti^3+^ (457.4 eV), were identified. Ti^3+^ is considered to originate from oxygen vacancies. Thus, we can infer that the reduction in this study proceeds via a similar mechanism when methanol is used as the solvent. Considering the heterogeneous nucleation and catalytic effect of BaTiO_3_, the primary synthesis route for BaTiO_3_/Ag nanocomposite particles is the reduction at the BaTiO_3_ surface.

Because Ag_2_O was reduced by both mechanical stirring and sonication in the presence of BaTiO_3_, the BaTiO_3_/Ag nanocomposite particles may have been formed via two reaction pathways: sonication-independent reactions and reactions promoted by the decomposition products of methanol generated under ultrasound. Based on the GC–MS results shown in Fig. 7 (b, c), the following reactions are presumed to have occurred.

First, the sonication-independent reactions are discussed. Because methanol, used as the solvent, has reducing properties, it is inferred that Ag^+^ and Ag*_n_*^+^ was reduced by methanol, as shown in Eq. (1), to produce metallic Ag and formaldehyde.

(1) $$\begin{array}{c} 2{\mathrm{Ag}}^{+}\left({\mathrm{Ag}}_{n}^{\;\;+}\right)+{\mathrm{CH}}_{3}\mathrm{OH}\to 2{\mathrm{Ag}}^{0}\left({\mathrm{Ag}}_{n}^{\;\;0}\right)+\mathrm{HCHO}+2H^{+} \end{array}$$

Formaldehyde was produced during the reaction (Eq. (1)) and also acted as a reducing agent; presumably it further reduces Ag^+^ and Ag*_n_*^+^ to metallic Ag and generated formic acid, as shown in Eq. (2).

(2) $$\begin{array}{c} 2{\mathrm{Ag}}^{+}\left({\mathrm{Ag}}_{n}^{\;\;+}\right)+\mathrm{HCHO}+H_{2}O\to 2{\mathrm{Ag}}^{0}\left({\mathrm{Ag}}_{n}^{\;\;0}\right)+\mathrm{HCOOH}+2H^{+} \end{array}$$

Formic acid also exhibits reducing properties and is considered to reduce Ag^+^ and Ag*_n_*^+^ (Eq. (3)). Additionally, based on the results shown in Fig. 7 (b) and (c), methyl formate was also detected. Methyl formate was presumed to be generated through a reaction between formic acid and methanol present in the solution (Eq. (4)).

(3) $$\begin{array}{c} 2{\mathrm{Ag}}^{+}\left({\mathrm{Ag}}_{n}^{\;\;+}\right)+\mathrm{HCOOH}\to 2{\mathrm{Ag}}^{0}\left({\mathrm{Ag}}_{n}^{\;\;0}\right)+2H^{+}+{\mathrm{CO}}_{2} \end{array}$$

(4) $$\begin{array}{c} \mathrm{HCOOH}+{\mathrm{CH}}_{3}\mathrm{OH}\to {\mathrm{HCOOCH}}_{3}+H_{2}O \end{array}$$

In addition to the above reactions, methanol decomposition reactions also occurred under ultrasound irradiation. The decomposition of alcohols under ultrasound irradiation has been extensively studied [[44], [45], [46], [47]]. In particular, for water–methanol systems, ultrasound irradiation leads to the formation of methanol-decomposition products, as shown in Eq. (5) [44]:

(5) $$\begin{array}{c} {\mathrm{CH}}_{3}\mathrm{OH}+\mathrm{ultrasound}\to \mathrm{CO},\;H_{2},\;{\mathrm{CH}}_{4},\;\mathrm{HCHO},\;\mathrm{etc}. \end{array}$$

The decomposition products in Eq. (5) are formed via radical intermediates derived from methanol, as shown in Eq. (6):

(6) $$\begin{array}{c} {\mathrm{CH}}_{3}\mathrm{OH}+\mathrm{ultrasound}\to \cdot H,\;\cdot {\mathrm{CH}}_{2}\mathrm{OH},\;\cdot {\mathrm{CH}}_{3},\;\cdot \mathrm{OH},\mathrm{etc}. \end{array}$$

The reducing species and radicals in Eqs. (5), (6) were presumed to reduce the Ag precursor to metallic Ag (Eq. (7)).

(7) $$\begin{array}{c} {\mathrm{Ag}}^{+}\left({\mathrm{Ag}}_{n}^{\;\;+}\right)+\mathrm{reducing}\;\mathrm{agent}\to {\mathrm{Ag}}^{0}\left({\mathrm{Ag}}_{n}^{\;\;0}\right) \end{array}$$

In this study, ultrasonic irradiation was used under ambient-air conditions. Formic acid is generated when ultrasound irradiation is applied to a water–methanol system in the presence of oxygen [48]. Therefore, the formaldehyde and formic acid generated by ultrasound irradiation also contributed to the reactions shown in Eqs. (2), (3), (4). As shown in Fig. 7 (b) and (c), ethanol formation was also observed. Ethanol was assumed to be produced by the radical species generated from methanol. Ethanol is also a reducing alcohol and is presumed to exhibit a similar effect to methanol. However, its reducing ability is weaker than that of methanol. In practice, when the same synthesis was carried out using ethanol, the time required for complete reduction of Ag_2_O became 12 h, which was markedly slower (Fig. S11). In addition, the amount of ethanol was much smaller than that of methanol. Therefore, ethanol is presumed to contribute little to the reaction.

In the third stage, the growth of the Ag NPs was induced by ultrasound irradiation. It is presumed that this growth has taken place since the initial stage of Ag NP deposition. In general, NPs exhibit a low melting point and high diffusivity because of their high surface energies [49,50]. Given these characteristics of the Ag NPs, their growth is likely driven by thermal and physical effects induced by hotspots, shock waves, and microjet flows. In addition to the dissolution-reprecipitation mechanism (Ostwald ripening), sintering is also presumed to contribute to the growth of Ag NPs. Noble metal-supported catalysts undergo aggregation of noble metal NPs when exposed to high-temperature environments [51,52]. The “metal-migration model,” in which noble-metal NPs migrate on the support surface, has been proposed as a mechanism for aggregation [53,54]. Notably, the phenomena observed in this study differ significantly from those observed for supported noble-metal catalysts because the reactions occur in solution and the ultrasonic hotspots are highly localized and transient. However, the thermal and physical effects of ultrasound may cause the Ag NPs to move on the BaTiO_3_ surface and aggregate.

Finally, the effects of ultrasonic frequency on the reduction rate of Ag_2_O and morphology of Ag NPs are discussed. Chemical effects such as radical generation are promoted at high frequencies [9,55,56]. Based on the results shown in Fig. 1 and Fig. S4, no differences were observed in the reduction time of Ag_2_O. Therefore, the difference in the generation of radical species did not affect the reduction rate of Ag_2_O. Regarding the morphology, aggregation of Ag NPs was observed from the early stage of the reaction at 24 kHz (Fig. 3 and Fig. S5). Based on the relative comparison of cavitation intensity (Table S2), the intensity decreased in the order of 45, 24, and 100 kHz. Despite having the second-lowest cavitation intensity, the most aggregated Ag NPs were observed at 24 kHz. This result indicates that the aggregation observed at 24 kHz is not primarily caused by sintering due to physical effects. Cavitation intensity has been reported to influence the aggregation process, with stronger cavitation enhancing the disaggregation effect [[57], [58], [59]]. Since 24 kHz produces lower cavitation intensity than 45 kHz, the effect of disaggregation was reduced, which likely contributed to partial aggregation of Ag NPs. Although 100 kHz provides the lowest cavitation intensity, ultrasound irradiation with higher frequency generates a greater number of bubbles [9], thereby promoting nucleation and resulting in the deposition of fine Ag NPs. In contrast, at 24 kHz, the reduced bubble population not only suppressed nucleation but also caused non-uniformly nucleation due to coarse, transient cavitation [60], leading to aggregation from the early stages of the reaction. Moreover, bubbles at 24 kHz are the largest, and their collapse produces the strongest cavitation intensity per bubble. Consequently, local sintering or growth effects may have occurred, further promoting the aggregation of Ag NPs.

## 4 Conclusion

In this study, we synthesized BaTiO_3_/Ag nanocomposite particles using a sonochemical process. The ultrasound frequency and the Ag volume fraction were also investigated. In addition, we examined the mechanism of Ag NPs deposition on BaTiO_3_ particles. The following conclusions were drawn:

• BaTiO_3_/Ag nanocomposite particles with fine (∼20 nm) and uniformly deposited Ag NPs were synthesized after 3 h of ultrasound irradiation (>45 kHz).
• Even at a high Ag loading (10 vol%), Ag NPs were successfully deposited without a dispersant. Samples with 5 vol% Ag or less showed absorption owing to the SPR of Ag NPs, but their wavelength differed from that of conventional Ag NPs.
• Compared with mechanical stirring, ultrasound irradiation promoted the reduction rate of Ag_2_O and contributed to the refinement and uniform deposition of Ag NPs. Based on the main mechanism of Ag-NP deposition, Ag NPs precursors (Ag^+^, Ag*_n_*^+^) were reduced by species derived from methanol (methanol, formaldehyde, formic acid, and reducing radicals) on the surface of BaTiO_3_. Ultrasound irradiation for more than 3 h caused the diffusion (sintering) and dissolution-reprecipitation of Ag NPs, which led to Ag NP growth.

This process is a simple method that enables the synthesis of nanocomposite particles via the irradiation of BaTiO_3_ and Ag_2_O in methanol. Moreover, the nanocomposite particles were synthesized at room temperature with minimal waste from the precursor salt and dispersant. The growth of Ag NPs under ultrasound irradiation indicates the possibility of controlling the particle size via ultrasound irradiation. Nanomaterials have been limited in practical applications owing to their high cost, and environmental impact owing to the generation of large amounts of waste during synthesis, and handling difficulties. However, the proposed approach is expected to be an eco-friendly process that can help address these challenges. This approach is also expected to be applicable to the synthesis of various noble-metal NP/ceramic nanocomposite particles by using other noble metal oxides and ceramic particles. Energy efficiency can be further improved through optimization of the ultrasound irradiation device. This simple and straightforward process is expected to be easier to scale up compared with previous complicated processes. To advance industrial applications, future research should prioritize the development and investigation of large-scale ultrasound irradiation systems.

## CRediT authorship contribution statement

**Tatsuya Shishido:** Writing – original draft, Investigation, Formal analysis, Conceptualization. **Yamato Hayashi:** Writing – review & editing, Project administration, Methodology, Investigation, Conceptualization. **Hirotsugu Takizawa:** Supervision. **Minoru Ueshima:** Project administration.

## Declaration of competing interest

The authors declare that they have no known competing financial interests or personal relationships that could have appeared to influence the work reported in this paper.
