# Supplementary material for: Eco-friendly sonochemical synthesis of BaTiO3/Ag nanocomposite particles: Investigation of Ag-nanoparticle formation and growth behavior under ultrasound irradiation
Source: Ultrason Sonochem. 2025 Aug 28;121:107539. doi: 10.1016/j.ultsonch.2025.107539 (PMC12444157; doi:10.1016/j.ultsonch.2025.107539)
Supplement: Supplementary Data 1 [file mmc1.docx]

**Supporting Information**

**Eco-friendly sonochemical synthesis of BaTiO_3_/Ag nanocomposite particles: Investigation of Ag-nanoparticle formation and growth behavior under ultrasound irradiation**

Tatsuya Shishido^a^, Yamato Hayashi^a^, Hirotsugu Takizawa^a^, Minoru Ueshima^b^

^a^ Graduate School of Engineering, Department of Applied Chemistry, Tohoku University, 6-6-07 Aoba, Aramaki, Aoba-ku, Sendai 980-8579, Japan

^b^ Daicel Corporation, Grand Front Osaka Tower-B, 3-1, Kita-ku, Osaka, 530-0011, Japan

Corresponding author: Y. Hayashi; E-mail address: yamato.hayashi.b6@tohoku.ac.jp

Table S1 Methods and characteristic for synthesis of BaTiO_3_/Ag nanocomposite particles.

| No. | Method | Starting Materials | Temp. (˚C) | The size of Ag NPs (nm) | Ref. |
| --- | --- | --- | --- | --- | --- |
| 1 | Chemical precipitation | AgNO_3_, H_2_O, NaOH | 85 | -50 | [1] |
| 2 | Polyol | AgNO_3_ , EG, PVP | 160 | 3-15 | [2] |
| 3 | Photoreduction | AgNO_3_, H_2_O | R.T. | 5-10 | [3] |
| 4 | Chemical reduction | AgNO_3_, NH_3_ aq., Glucose aq. | R.T. | 25 | [4] |
| 5 | Photoreduction | AgNO_3_, H_2_O | R.T. | 6 | [5] |
| 6 | Chemical precipitation | AgNO_3_, H_2_O, NaOH | R.T. | 20-30 | [6] |
| 7 | Polyol | AgNO_3_, EG | 140 | 5-20 | [7] |
| 8 | Chemical reduction | AgNO_3_, DA-HCl, H_2_O, NH_3_ aq. | R.T. | 10-45 | [8] |

- EG: ethylene glycol, PVP: Polyvinylpyrrolidone, DA-HCl: Dopamine hydrochloride


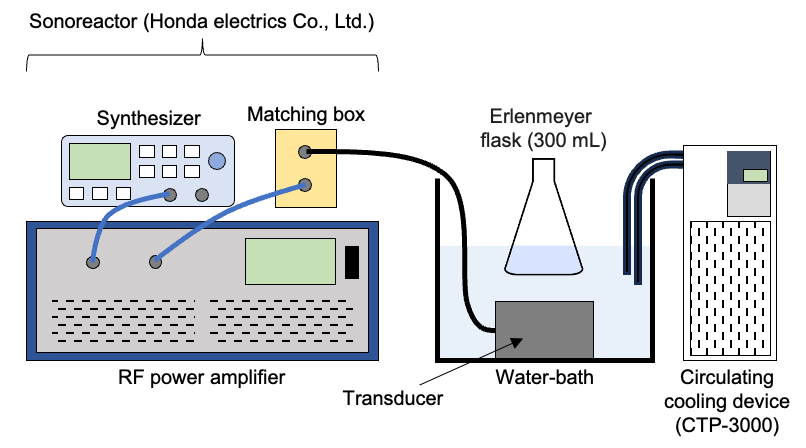


Fig. S1 The scheme of ultrasound reaction system.

Table S2 Input power and cavitation intensity at different frequencies.

| Frequency (kHz) | Input power (W) | Cavitation intensity (mA) | Energy efficiency (-) |
| --- | --- | --- | --- |
| 24 | 139 | 6 | 0.079 |
| 45 | 100 | 16 | 0.11 |
| 100 | 97.0 | 3 | 0.11 |

- Energy efficiency was calculated by *P*_ac_/Input power.


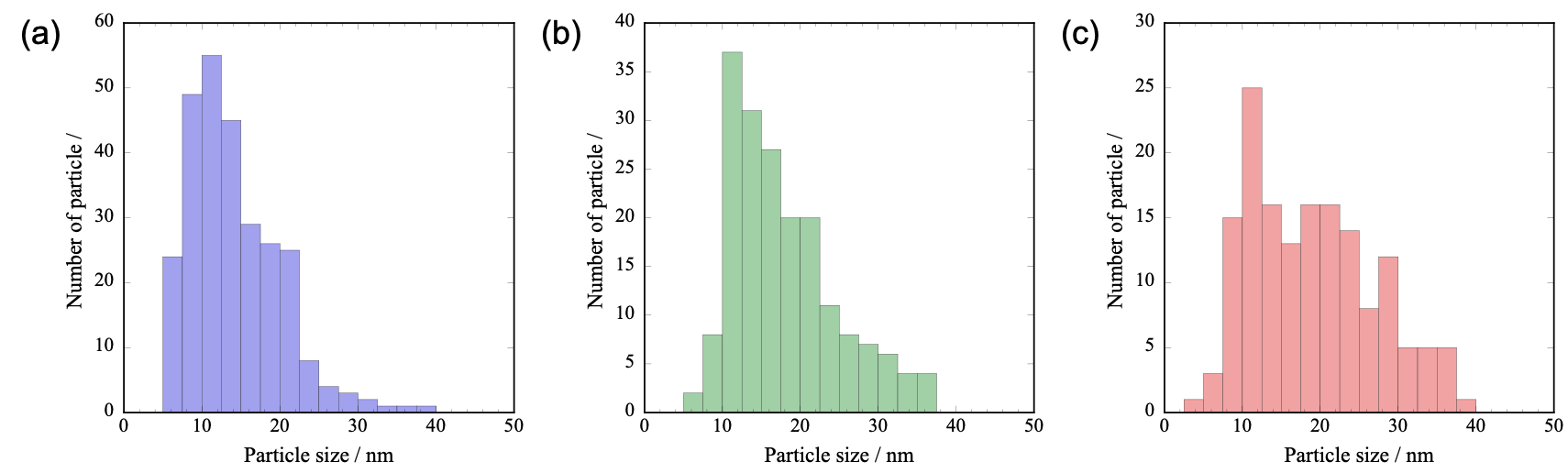


Fig. S2 Particle size distribution of deposited Ag NPs prepared by 45 kHz irradiation (Ag 5 vol%). (a) 3 h, (b) 12 h and (c) 24 h.

Table S3 Summary of statistical data of deposited Ag NPs synthesized by 45 kHz irradiation (Ag 5 vol%).

|  | 3 h | 12 h | 24 h |
| --- | --- | --- | --- |
| *d*_ave_ (nm) | 14.1 | 17.8 | 18.8 |
| Standard deviation (nm) | 5.9 | 6.8 | 8.0 |
| *d*_50_ (nm) | 12.9 | 16.0 | 17.8 |


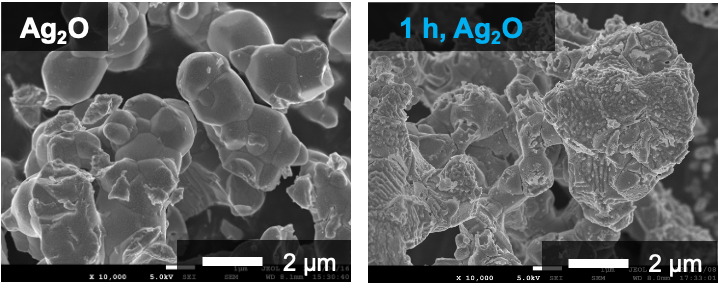


Fig. S3 SEM images of (a) Ag_2_O and (b) residual Ag_2_O after ultrasound irradiation.


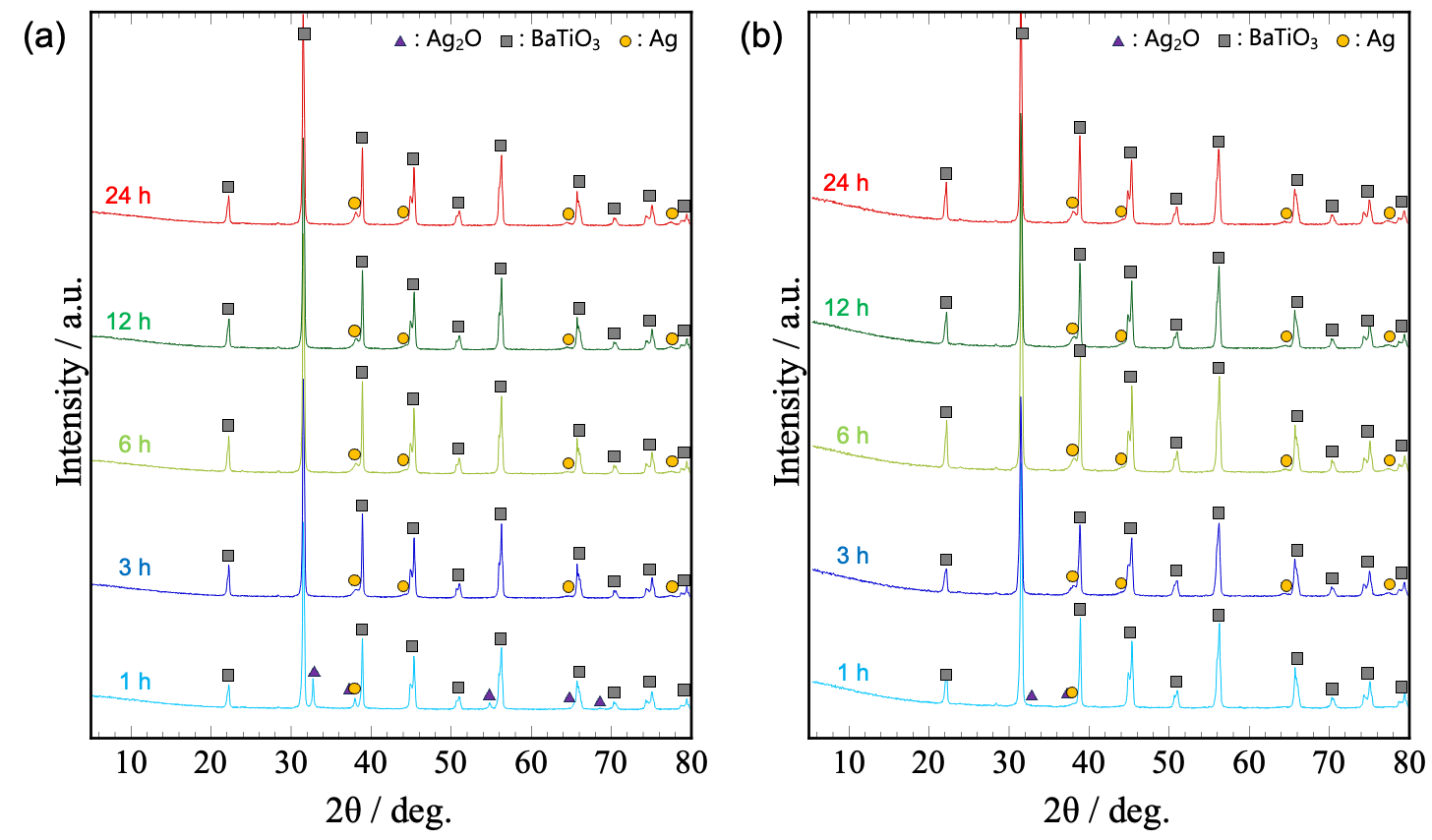


Fig. S4 XRD patterns of samples. (a) 24 kHz and (b) 100 kHz irradiation.


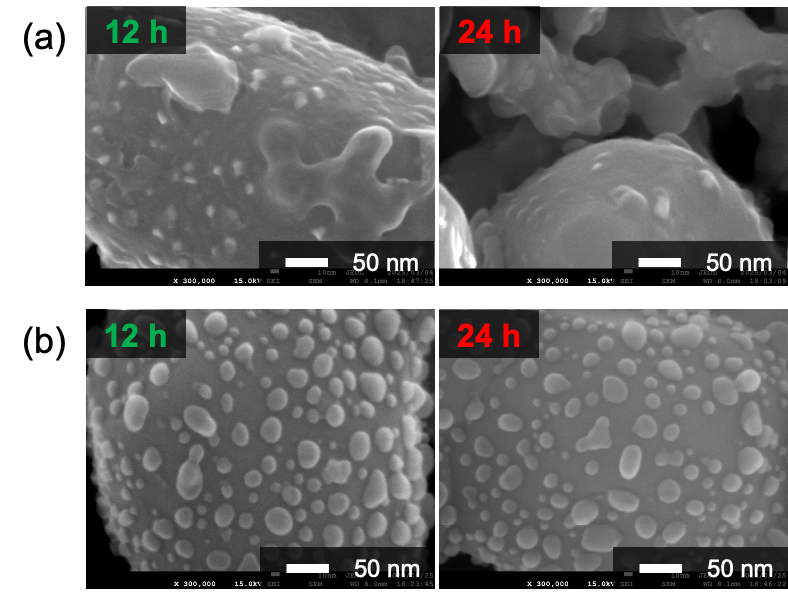


Fig. S5 SEM images of samples. (a) 24 kHz and (b) 100 kHz irradiation.


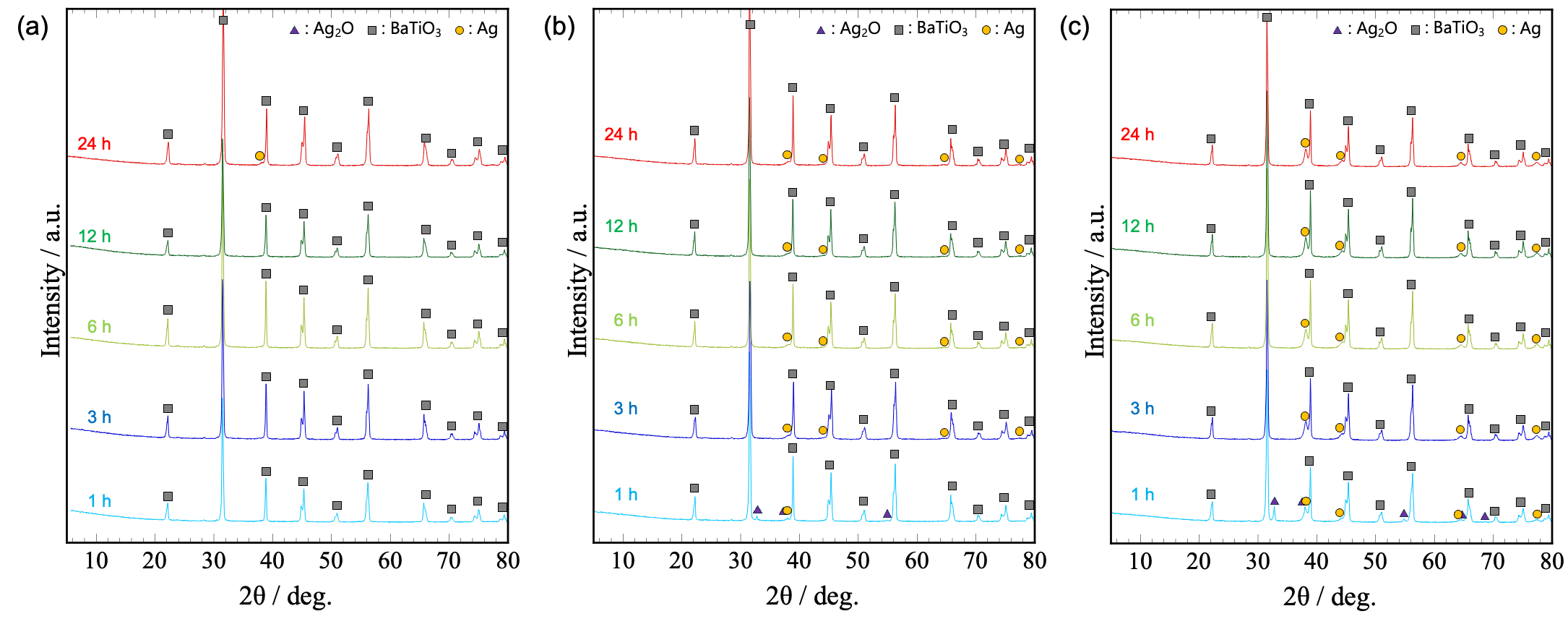


Fig. S6 XRD patterns of samples. (a) Ag 1 vol%, (b) Ag 3 vol% and (c) Ag 10 vol%.


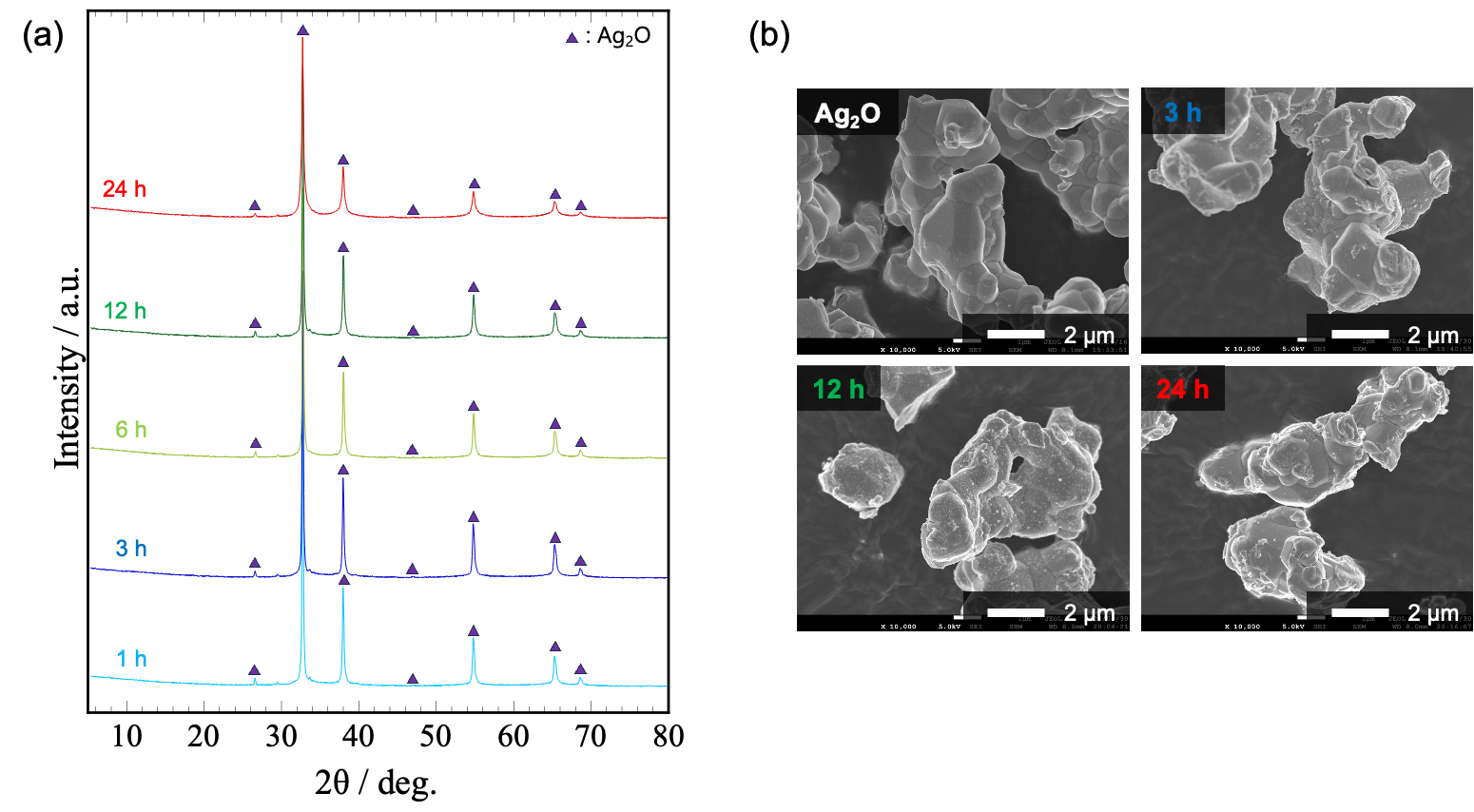


Fig. S7 (a) XRD pattens and (b) SEM images of Ag_2_O after mechanical stirring.

The reaction rate in ultrasound irradiation and mechanical stirring was estimated using the following equation.

$$\ln\frac{\left[ Ag_{2}O \right]_{t}}{\left[ Ag_{2}O \right]_{0}}=-kt$$

where $\left[ Ag_{2}O \right]_{0}$ and $\left[ Ag_{2}O \right]_{t}$ correspond to the concentration of Ag_2_O at times *t* and *t* = 0 (original concentration of Ag_2_O), respectively. $\left[ Ag_{2}O \right]_{t}$ was calculated by TG-TDA results, which showed weight loss due to thermal decomposition of Ag_2_O.


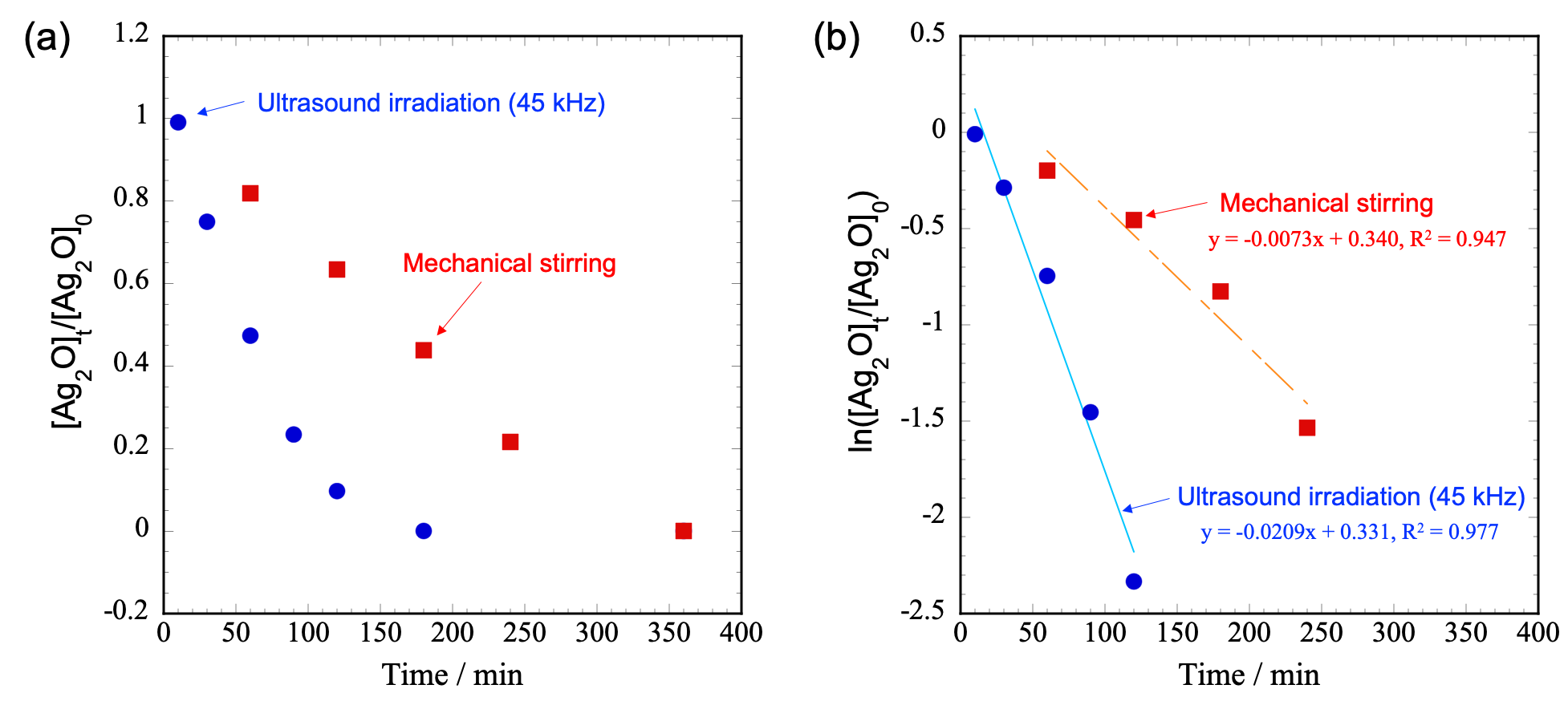


Fig. S8 (a) Time dependence of Ag_2_O conversion rate, (b) Linear fit of the kinetic curve.


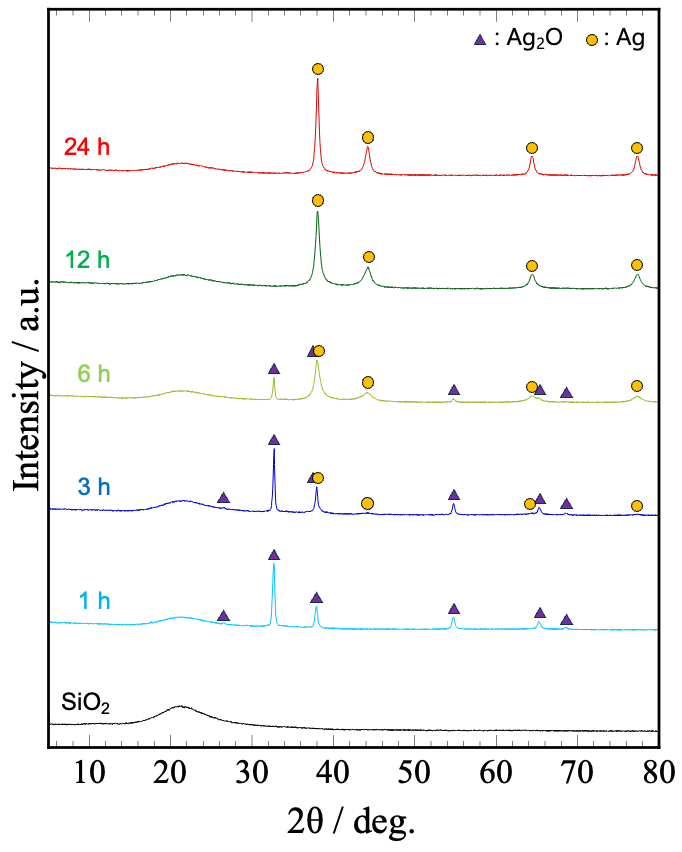


Fig. S9 Control experiment for BaTiO_3_ catalytic activity.


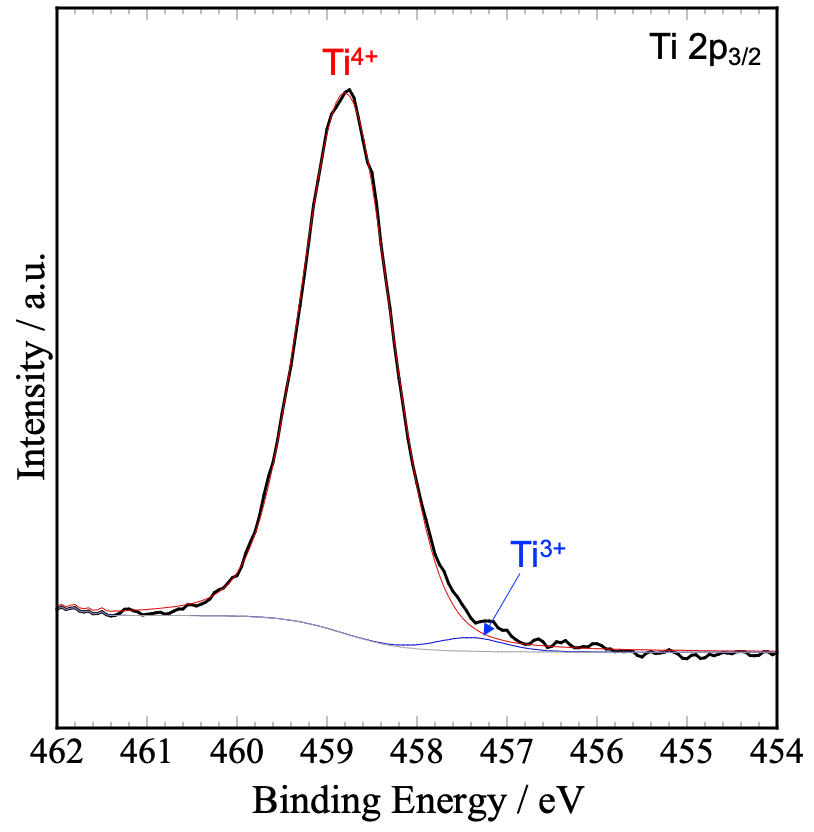


Fig. S10 Ti 2p XPS spectrum of BaTiO_3_.


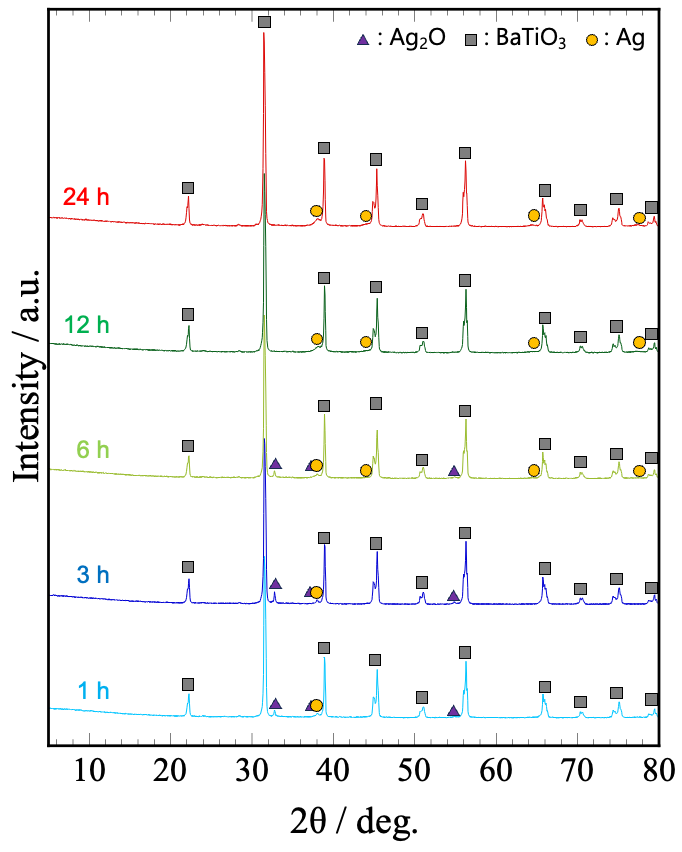


Fig. S11 XRD pattens of samples prepared by ethanol.

**Reference**

[1] Z. Hu, W. Dong, Z. Dong, P. Li, Q. Bao, T. Cao, G. Zhao, Low temperature one-step synthesis of Ag nanoparticles on BaTiO_3_ for synergistic piezo-photocatalytic properties, Colloids and Surfaces A: Physicochemical and Engineering Aspects 691 (2024) 133948. https://doi.org/10.1016/j.colsurfa.2024.133948.

[2] L. Xie, X. Huang, B.-W. Li, C. Zhi, T. Tanaka, P. Jiang, Core – satellite Ag@BaTiO_3_ nanoassemblies for fabrication of polymer nanocomposites with high discharged energy density, high breakdown strength and low dielectric loss, Phys. Chem. Chem. Phys. 15 (2013) 17560 – 17569. https://doi.org/10.1039/C3CP52799A.

[3] Y. Cui, J. Briscoe, S. Dunn, Effect of ferroelectricity on solar-light-driven photocatalytic activity of BaTiO_3_ – Influence on the carrier separation and stern layer formation, Chem. Mater. 25 (2013) 4215 – 4223. https://doi.org/10.1021/cm402092f.

[4] T. Kojima, M. Sugihara, Y. Hosoi, N. Uekawa, K. Kakegawa, Fabrication of BaTiO_3_/Ag composites using uniform Ag-deposited BaTiO_3_ particles, J. Ceram. Soc. Jpn. 117 (2009) 1328 – 1332. https://doi.org/10.2109/jcersj2.117.1328.

[5] S. Xu, Z. Liu, M. Zhang, L. Guo, Piezotronics enhanced photocatalytic activities of Ag-BaTiO_3_ plasmonic photocatalysts, J. Alloys Compd. 801 (2019) 483 – 488. https://doi.org/10.1016/j.jallcom.2019.06.115.

[6] H. Gao, Y. Han, Y. Wang, H. Xia, X. Zhu, D. Wang, Y. Zhang, X. Mao, L. Zhang, Dissolved oxygen enhanced piezo-photocatalytic performance in Ag dots-modified BaTiO_3_ nanoparticles for efficient degradation of multiple organic pollutants, Sep. Purif. Technol. 346 (2024) 127548. https://doi.org/10.1016/j.seppur.2024.127548.

[7] S. Luo, S. Yu, R. Sun, C.-P. Wong, Nano Ag-deposited BaTiO_3_ hybrid particles as fillers for polymeric dielectric composites: Toward high dielectric constant and suppressed loss, ACS Appl. Mater. Interfaces 6 (2014) 176 – 182. https://doi.org/10.1021/am404556c.

[8] C. Shuai, G. Liu, Y. Yang, F. Qi, S. Peng, W. Yang, C. He, G. Wang, G. Qian, A strawberry-like Ag-decorated barium titanate enhances piezoelectric and antibacterial activities of polymer scaffold, Nano Energy 74 (2020) 104825. https://doi.org/10.1016/j.nanoen.2020.104825.
